# Composition and Predicted Metabolic Capacity of Upper and Lower Airway Microbiota of Healthy Dogs in Relation to the Fecal Microbiota

**DOI:** 10.1371/journal.pone.0154646

**Published:** 2016-05-02

**Authors:** Aaron C. Ericsson, Alexa R. Personett, Megan E. Grobman, Hansjorg Rindt, Carol R. Reinero

**Affiliations:** 1 University of Missouri Metagenomics Center, University of Missouri, Columbia, Missouri, United States of America; 2 College of Veterinary Medicine, University of Missouri, Columbia, Missouri, United States of America; 3 Department of Veterinary Pathobiology, College of Veterinary Medicine, University of Missouri, Columbia, Missouri, United States of America; 4 Department of Veterinary Medicine and Surgery, College of Veterinary Medicine, University of Missouri, Columbia, Missouri, United States of America; 5 Comparative Internal Medicine Laboratory, University of Missouri, Columbia, Missouri, United States of America; University of Illinois at Urbana-Champaign, UNITED STATES

## Abstract

The upper and lower airways of healthy humans are reported to harbor stable and consistent bacterial populations, and the composition of these communities is altered in individuals affected with several respiratory diseases. Data regarding the presence of airway microbiota in other animals are scant and a better understanding of the composition and metabolic function of such bacterial populations is essential for the development of novel therapeutic and diagnostic modalities for use in both veterinary and human medicine. Based on targeted next-generation sequencing of feces and samples collected at multiple levels of the airways from 16 healthy female dogs, we demonstrate that canine airways harbor a topographically continuous microbiota with increasing relative abundance of proteobacterial species from the upper to lower airways. The lung-associated microbiota, as assessed via bronchoalveolar lavage fluid (BALF), was the most consistent between dogs and was dominated by three distinct taxa, two of which were resolved to the species level and one to the level of family. The gene content of the nasal, oropharyngeal, and lung-associated microbiota, predicted using the Phylogenetic Investigations into Communities by Reconstruction of Unobserved States (PICRUSt) software, provided information regarding the glyoxylate and citrate cycle metabolic pathways utilized by these bacterial populations to colonize such nutrient-poor, low-throughput environments. These data generated in healthy subjects provide context for future analysis of diseased canine airways. Moreover, as dogs have similar respiratory anatomy, physiology, and immune systems as humans, are exposed to many of the same environmental stimuli, and spontaneously develop similar respiratory diseases, these data support the use of dogs as a model species for prospective studies of the airway microbiota, with findings translatable to the human condition.

## Introduction

The influence on host health of the complex and dynamic community of microbes present in the gastrointestinal tract, i.e., the gut microbiota, has become a rapidly expanding area of biomedical research. Of all of the anatomic sites investigated in the National Institutes of Health’s Human Microbiome Project (HMP)[1], the gut is undoubtedly the most widely studied. Interestingly, the respiratory tract was not included in the HMP, partially owing to the long-held belief that the airways, and particularly the lower airways, were largely devoid of colonizing microbial populations and any bacteria detected there were either transient or the result of defective clearance mechanisms. This belief was based on the lack of discernible colonization via histological examination and negative results using traditional culture-based microbiological methods. With the development of culture-independent molecular methods to characterize complex microbial communities, it has become clear that healthy airways do contain resident bacterial populations [2, 3]. While the influence of these microbes on the development and function of the immune system, colonization resistance against potential pathogens, and overall respiratory function is unclear, several groups have documented changes in the community structure of those microbes in a multitude of inflammatory conditions affecting the human respiratory system including cystic fibrosis [4], chronic obstructive pulmonary disease [2, 5], and asthma [6, 7]. As in the gut, whether such changes in microbial composition represent examples of dysbiosis of the airway microbiota contributing to the disease pathogenesis, or are simply reactions to altered host physiology is not clear.

The majority of research investigating the airway microbiota has been performed in humans, and very little is known regarding the presence or composition of similar bacterial populations in other species. An increased understanding of the airway microbiota in companion animal species would provide several benefits. First, respiratory diseases are common in companion animal veterinary medicine, typified by inflammatory airway disorders such as canine chronic bronchitis and feline asthma. After food, health care represents the largest overall cost to pet-owners with over $15 billion being spent on veterinary care in 2014, according to the American Pet Products Association. Additionally, almost every diagnostic modality or treatment available in human patients is also employed in veterinary medicine (including bacteriotherapy), suggesting that such information would be of immediate interest and use to veterinary practitioners. Second, and perhaps more importantly, there is a need for animal models of human respiratory disease and there is reason to believe that companion animals such as dogs and cats may be ideal host species. The bronchopulmonary anatomy of large dogs is of a similar size to that of adult humans, while that of small dogs and cats is of a similar size to pediatric human patients. Similarly, as companion animals, they are exposed to many of the same environmental exposures, and subject to many of the same disease processes as humans including, for example, feline allergic asthma [8].

To determine if consistent airway microbial communities are present in healthy dogs, samples were collected from multiple levels of the upper (nasal and oropharyngeal) and lower (pulmonary) airways of 16 healthy dogs, and subjected to rigorous DNA extraction methods, followed by 16S rRNA amplicon sequencing. Concomitant fecal samples were also collected and similarly processed to investigate possible correlations in the composition of microbiota detected at each airway sample site. Lastly, predicted gene content of the bacterial communities at each site was used to infer key metabolic pathways and energy sources of the airway microbiota of healthy dogs.

## Results

### Complex and Robust Bacterial Communities Are Detected at All Sample Sites

Ideally, a normalized amount of 100 ng of total DNA is used as template in the amplification of the V4 region of the 16S rRNA gene prior to sequencing. Based on fluorometric measurements using a broad-range dsDNA kit, optimal DNA yields were obtained from all 16 fecal samples (mean ± SEM of 14.9 ± 3.5 ng/μL), 15 of 16 nasal swabs (55.7 ± 9.8 ng/μL), and 3 of 16 oropharyngeal swab samples (11.5 ± 4.6 ng/μL in the 13 samples that were above the lower limit of detection). Bronchoalveolar lavage fluid was much more dilute and no samples yielded 100 ng of DNA; the majority of samples were below the lower limit of detection. To maximize the amount of loading template for those samples providing scant DNA, samples were concentrated to the minimal volume required for PCR and no further measurements were taken. All other samples yielding adequate DNA were normalized to a standard concentration for PCR.

Following amplification in a 96-well format using single-indexed primers, samples were pooled for sequencing in a single lane using the Illumina MiSeq platform. Sequencing coverage of the samples from the four sample sites varied substantially with significantly greater numbers of sequence reads generated from fecal samples relative to all other sites (S1A Fig), despite normalization of loading template in the majority of nasal swab samples. As anticipated, coverage for the BALF samples was the lowest of all sample sites (mean ± SEM of 5706 ± 997 sequences/sample), although consistent and interpretable data were generated for all samples.

Surprisingly, annotation of sequence data revealed coverage-independent differences in microbial richness between sample sites, with subjectively greater numbers of operational taxonomic units (OTUs) detected in upper airway samples relative to feces and BALF (S1B Fig). This difference achieved significance in the case of oropharyngeal swabs, as compared to feces and BALF (Kruskal-Wallis one way ANOVA on ranks with post hoc pairwise multiple comparisons via Tukey test).

### Different Complex Microbiota Are Specific to Each Sample Site

**Fig 1:**
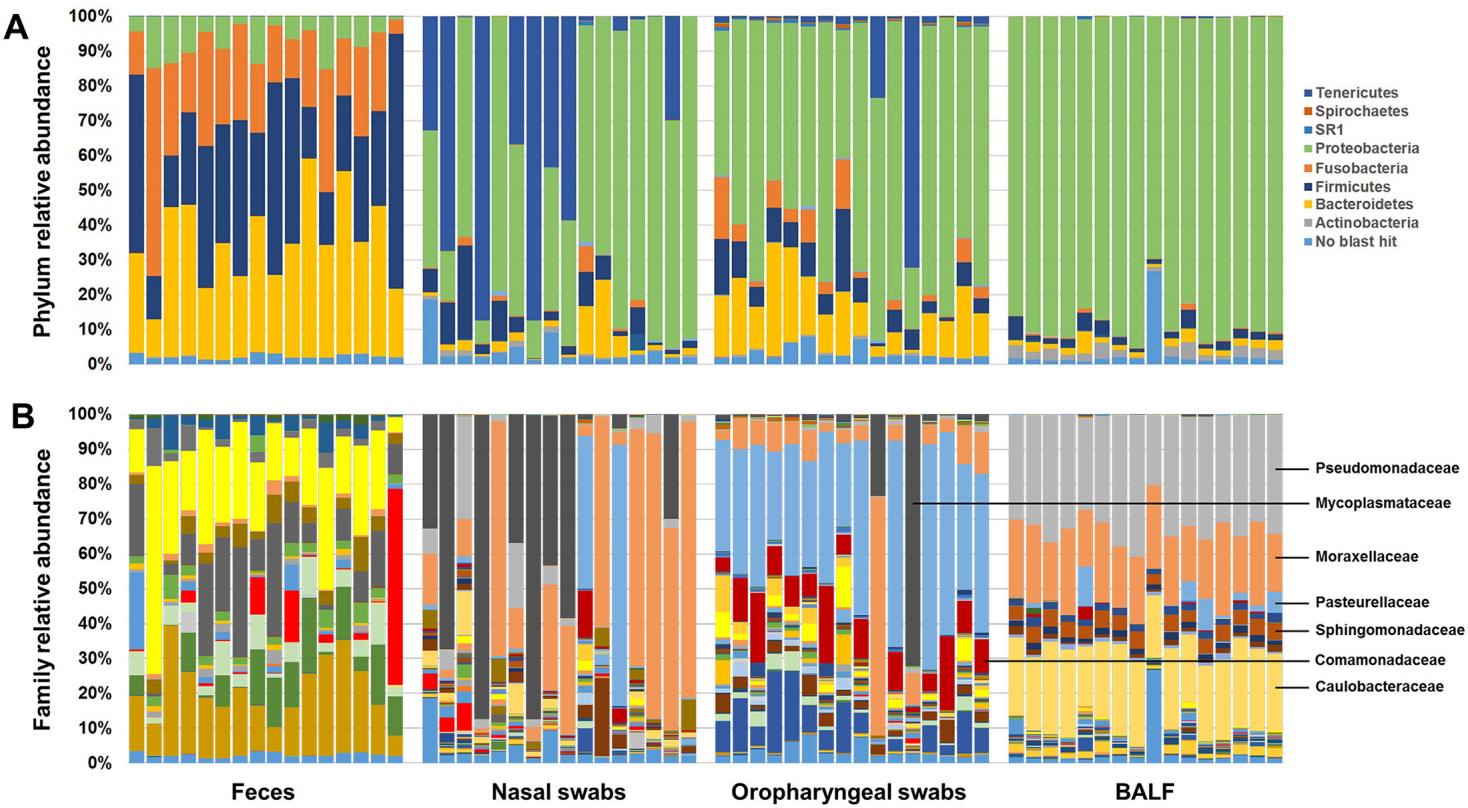
Phylum- and family-level composition of fecal and airway microbiota. Bar charts showing relative abundance of all taxa detected in feces, nasal swabs, oropharyngeal swabs, or bronchoalveolar lavage fluid (BALF) collected from 16 intact adult female dogs, annotated to the taxonomic level of phylum (**A**) or family (**B**). Legend for all detected phyla is shown at upper right; identity of selected microbial families consistently detected in airway samples are shown at bottom right. Samples are shown in the same order, within each collection site, i.e., the first bar from each sample site was collected from the same dog.

Comparison of the detected microbial profiles at the taxonomic level of phylum indicated a clear difference between feces and airway samples (Fig 1A). In agreement with previous reports [9], fecal samples were dominated by the four phyla *Bacteroidetes* (mean ± SEM relative abundance of 33.43 ± 3.14%), *Firmicutes* (33.38 ± 4.36%), *Fusobacteria* (23.21 ± 3.15%), and *Proteobacteria* (7.58 ± 1.19%). Upper and lower airway samples were dominated by the phylum *Proteobacteria* (55.40 ± 7.14%, 60.79 ± 4.67%, and 88.53 ± 1.56% in nasal, oropharyngeal, and BALF samples respectively), although the other three phyla were also detected in all samples. Bacteria in the phylum *Tenericutes* were also detected in all fecal and oropharyngeal samples (0.014 ± 0.003% and 7.04 ± 4.56% respectively), and all but one nasal swabs (28.08 ± 8.11%). Notably, while *Tenericutes* represented a dominant phylum in approximately half of the nasal swabs and two of the oropharyngeal swabs, this phylum was detected in only 6 of 16 BALF samples (at very low levels), suggesting that this phylum preferentially colonizes the upper airways but is much less common in the lungs. Conversely, the phylum *Actinobacteria*, detected in all samples from all sites, appeared to preferentially colonize the lungs, with mean ± SEM relative abundance of 0.07 ± 0.03%, 0.73 ± 0.16%, 0.41 ± 0.07%, and 2.61 ± 0.32% in feces, nasal, oropharyngeal, and BALF samples respectively.

Resolved to the taxonomic level of family, compositional differences between the sample sites, particularly between feces and airway samples, became more apparent (Fig 1B). The phylum *Bacteroidetes*, detected at a greater relative abundance in feces and oropharyngeal samples relative to nasal swabs and BALF, comprised different bacterial families and OTUs. In feces, phylum *Bacteroidetes* consisted primarily of microbes in the families *Bacteroidaceae* (18.78 ± 2.24%), *Prevotellaceae* (8.73 ± 1.79%), and *Paraprevotellaceae* (5.36 ± 0.85%) whereas in oropharyngeal samples, this phylum represented primarily *Porphyromonadaceae* (8.76 ± 1.69%) with only scant amounts of the feces-associated families. Similarly, the relatively low levels of microbes in the phylum *Proteobacteria* detected in feces comprised primarily the families *Succinivibrionaceae* (class *Gammaproteobacteria*; 3.38 ± 0.76%), *Alcaligenaceae* (class *Betaproteobacteria*; 2.85 ± 0.68%), and *Helicobacteraceae* (class *Epsilonproteobacteria*; 0.40 ± 0.30%), while those taxa were extremely rare in all airway samples. Alternatively, class *Alphaproteobacteria* was much more prevalent in nasal and oropharyngeal swabs and BALF, with substantial levels of family *Caulobacteraceae* (2.71 ± 0.84%, 0.41 ± 0.09%, and 22.52 ± 0.68% respectively), and in the BALF particularly, *Sphingomonadaceae* (4.33 ± 0.16%), *Methylobacteriaceae* (1.68 ± 0.08%), and *Bradyrhizobiaceae* (1.23 ± 0.13%). Other prominent *Betaproteobacteria* in the nasal, oropharyngeal, and BALF samples included *Comamonadaceae* (0.32 ± 0.07%, 0.71 ± 0.31%, and 1.94 ± 0.09% respectively) and *Neisseriaceae* (1.44 ± 0.84%, 9.81 ± 1.65%, and 0.44 ± 0.22% respectively). Perhaps not surprisingly, airway samples were dominated by facultative and obligate aerobic families, including *Pasteurellaceae*, *Moraxellaceae*, and *Pseudomonadaceae*, in varying proportions. Nasal swabs were dominated by *Moraxellaceae* (33.00 ± 7.68%) with less *Pasteurellaceae* and *Pseudomonadaceae* (7.90 ± 5.22% and 5.57 ± 1.93% respectively); oropharyngeal swabs were dominated by *Pasteurellaceae* (36.49 ± 4.60%) with less *Moraxellaceae* and *Pseudomonadaceae* (10.36 ± 3.94% and 0.66 ± 0.14% respectively); and BALF had high relative abundance of both *Moraxellaceae* and *Pseudomonadaceae* (20.18 ± 0.92% and 32.38 ± 1.20% respectively) with less *Pasteurellaceae* (2.39 ± 0.90%). While it was highly variable between dogs, several nasal swabs and two oropharyngeal swabs also contained abundant DNA specific to the family *Mycoplasmataceae*.

**Fig 2:**
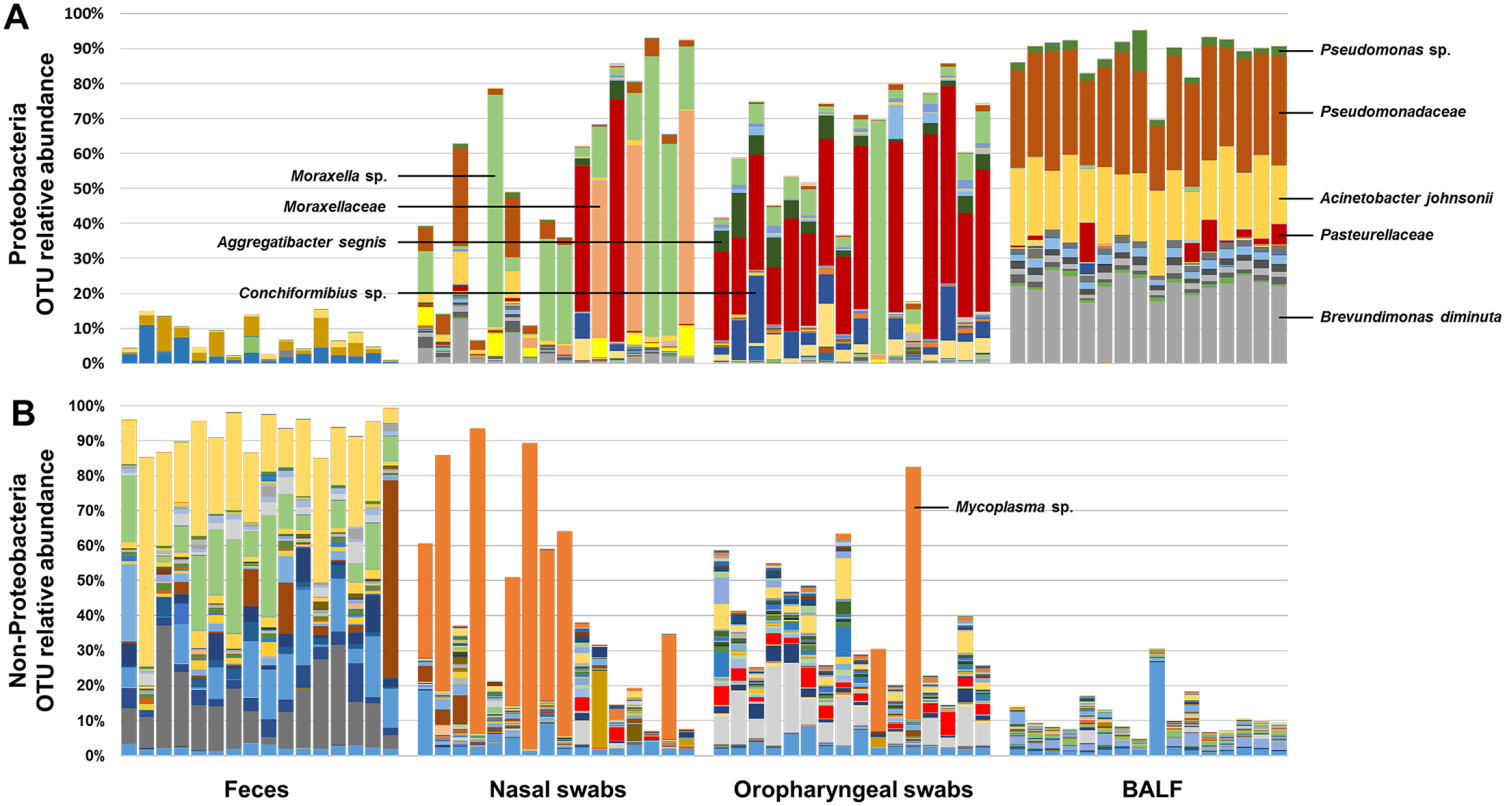
Operational taxonomic unit-level composition of fecal and airway microbiota. Bar charts showing relative abundance of operational taxonomic units (OTUs) in the phylum *Proteobacteria* (**A**) or all other phyla (**B**) detected in feces, nasal swabs, oropharyngeal swabs, or bronchoalveolar lavage fluid (BALF) collected from 16 intact adult female dogs. Predominant OTUs detected in airway samples are labeled. Samples are shown in the same order, within each collection site, i.e., the first bar from each sample site was collected from the same dog.

Resolved to the level of operational taxonomic unit (OTU), i.e., groups of sequences sharing a minimum of 97% nucleotide identity, the uniformity of the proteobacterial BALF-associated microbiota remained evident (Fig 2A). While the majority of DNA specific to family *Pseudomonadaceae* detected in the airway samples could not be resolved beyond the taxonomic level of family, DNA specific to *Pseudomonas* sp. constituted 0.33 ± 0.11%, 0.06 ± 0.01%, and 2.73 ± 0.61% in nasal, oropharyngeal, and BALF samples respectively. Low levels of DNA specific to *Pseudomonas thermotolerans* was also detected in 19% (3/16) of BALF samples. Interestingly, while the family *Moraxellaceae* represented a large portion of sequences detected in airway samples, the dominant OTUs within that family differed between upper and lower airways. Nasal and oropharyngeal swabs yielded a substantial relative abundance of *Moraxella* sp. (20.49 ± 6.36% and 8.57 ± 3.93% respectively), with much lower (but consistent) levels of *Acinetobacter* sp., *Acinetobacter johnsonii*, *Enhydrobacter* sp., and *Psychrobacter sanguinis*. There was also a sizeable proportion of sequences detected in nasal swabs that could not be resolved beyond the level of *Moraxellaceae* (10.31 ± 5.34%). Conversely, the majority of DNA specific to *Moraxellaceae* detected in BALF samples annotated to *Acinetobacter johnsonii*, with much lower relative abundance of the other aforementioned genera found in upper airways, including *Moraxella* sp. The prominent family *Caulobacteraceae* detected in BALF samples consisted almost entirely of one taxon, *Brevundimonas diminuta*.

Considering microbes outside of the phylum *Proteobacteria*, there were a limited number of taxa detected at a mean relative abundance greater than 0.50% including *Propionibacterium acnes* (1.90 ± 0.22% in BAL), *Porphyromonas* sp. (0.67 ± 0.42% and 8.32 ± 1.65% in nasal and oropharyngeal swabs respectively), *Prevotella* sp. (1.91 ± 0.42% in oropharyngeal swabs), *Flavobacterium* sp. (0.98 ± 0.09% in BALF), unresolved taxa in the family *Weeksellaceae* (0.61 ± 0.32% and 2.70 ± 0.48% in nasal and oropharyngeal swabs respectively), *Riemerella* sp. (1.51 ± 1.38% in nasal swabs), *Gemella* sp. and unresolved taxa in the family *Gemellaceae* (0.70 ± 0.18% and 0.51 ± 0.11% respectively in oropharyngeal swabs), *Lactobacillus* sp. (1.40 ± 0.55% in nasal swabs), *Streptococcus* sp. (1.06 ± 0.24, 0.72 ± 0.13%, and 0.85 ± 0.21% in nasal, oropharyngeal, and BALF samples respectively), *Clostridium* sp. (1.47 ± 0.64% in oropharyngeal swabs), *Fusobacterium* sp. (0.68 ± 0.22% and 2.96 ± 0.78% in nasal and oropharyngeal swabs respectively), unresolved taxa in the family *Leptotrichiaceae* (1.03 ± 0.47% in oropharyngeal swabs), and *Mycoplasma* sp. (27.99 ± 8.13% and 6.68 ± 4.59% in nasal and oropharyngeal swabs respectively) (Fig 2B). Interestingly, there was a trend toward these bacterial taxa being present at relatively greater abundance in either the upper or lower airways with only *Streptococcus* sp. being detected at greater than 0.50% (mean relative abundance) in both BALF and either nasal or oropharyngeal samples. Also of note, of the aforementioned taxa, 45% (5/11) of those detected at greater than 0.50% mean relative abundance exclusively in upper airway samples (i.e., *Porphyromonas* sp., *Prevotella* sp., *Lactobacillus* sp., *Clostridium* sp., and *Fusobacterium* sp.) were also detected in feces, while the two taxa detected at greater than 0.50% mean relative abundance exclusively in BALF samples (i.e., *Propionibacterium acnes* and *Flavobacterium* sp.) were not detected in feces. Table 1 shows the mean ± SEM relative abundance of all taxa detected at greater than 0.50% relative abundance in at least one sample site.

**Table 1:** Taxa present at > 0.50% mean relative abundance in one or more sample sites.

			Feces		Nasal swabs		OP swabs		BAL fluid	
*Phylum*	*Family*	OTU	mean	SEM	mean	SEM	mean	SEM	mean	SEM
*Actinobacteria*	*Propionibacteriaceae*	*Propionibacterium acnes*	0.00%	0.00%	0.34%	0.11%	0.14%	0.04%	1.90%	0.22%
*Bacteroidetes*	*Bacteroidaceae*	*Bacteroides* sp.	14.96%	2.21%	0.14%	0.03%	0.30%	0.05%	0.22%	0.05%
		*Bacteroides plebeius*	3.80%	0.63%	0.02%	0.01%	0.00%	0.00%	0.06%	0.01%
	*Porphyromonadaceae*	*Porphyromonas* sp.	0.02%	0.01%	0.67%	0.42%	8.32%	1.65%	0.44%	0.25%
	*Prevotellaceae*	*Prevotella copri*	8.72%	1.79%	0.23%	0.14%	0.02%	0.01%	0.09%	0.03%
	*[Paraprevotellaceae]*	Family [*Paraprevotellaceae*]	2.04%	0.41%	0.04%	0.01%	0.01%	0.00%	0.04%	0.01%
		[*Prevotella*] sp.	3.32%	0.85%	0.22%	0.13%	1.91%	0.42%	0.09%	0.04%
	*Flavobacteriaceae*	*Flavobacterium* sp.	0.00%	0.00%	0.08%	0.03%	0.01%	0.00%	0.98%	0.09%
	*[Weeksellaceae]*	Family [*Weeksellaceae*]	0.00%	0.00%	0.61%	0.32%	2.70%	0.48%	0.08%	0.04%
		*Riemerella* sp.	0.00%	0.00%	1.51%	1.38%	0.18%	0.18%	0.00%	0.00%
*Firmicutes*	*Gemellaceae*	Family *Gemellaceae*	0.00%	0.00%	0.18%	0.15%	0.70%	0.18%	0.03%	0.02%
		*Gemella* sp.	0.00%	0.00%	0.08%	0.06%	0.51%	0.11%	0.05%	0.02%
	*Lactobacillaceae*	*Lactobacillus* sp.	5.81%	3.53%	1.40%	0.55%	0.17%	0.10%	0.09%	0.02%
	*Streptococcaceae*	*Streptococcus* sp.	2.72%	1.37%	1.06%	0.24%	0.72%	0.13%	0.85%	0.21%
		Order *Clostridiales*	1.36%	0.26%	0.23%	0.11%	0.29%	0.12%	0.06%	0.03%
	*Clostridiaceae*	*Clostridium* sp.	0.44%	0.13%	0.08%	0.05%	1.47%	0.64%	0.03%	0.02%
	*Lachnospiraceae*	Family *Lachnospiraceae*	1.10%	0.14%	0.18%	0.06%	0.60%	0.13%	0.06%	0.02%
		*Dorea* sp.	0.59%	0.12%	0.07%	0.04%	0.00%	0.00%	0.02%	0.01%
		[*Ruminococcus*] *gnavus*	0.50%	0.13%	0.16%	0.12%	0.01%	0.01%	0.00%	0.00%
	*Ruminococcaceae*	Family *Ruminococcaceae*	2.00%	0.31%	0.06%	0.02%	0.01%	0.00%	0.02%	0.01%
		*Faecalibacterium prausnitzii*	11.23%	2.30%	0.25%	0.12%	0.01%	0.00%	0.03%	0.01%
	*Veillonellaceae*	*Megamonas* sp.	2.50%	0.46%	0.20%	0.11%	0.01%	0.00%	0.00%	0.00%
		*Phascolarctobacterium* sp.	1.18%	0.10%	0.05%	0.02%	0.00%	0.00%	0.00%	0.00%
		*Veillonella* sp.	0.72%	0.26%	0.03%	0.02%	0.01%	0.01%	0.00%	0.00%
	*[Tissierellaceae]*	*Parvimonas* sp.	0.00%	0.00%	0.08%	0.04%	0.58%	0.20%	0.03%	0.01%
	*Fusobacteriaceae*	*Fusobacterium* sp.	23.20%	3.15%	0.68%	0.22%	2.96%	0.78%	0.37%	0.09%
	*Leptotrichiaceae*	Family *Leptotrichiaceae*	0.00%	0.00%	0.11%	0.10%	1.03%	0.47%	0.00%	0.00%
		*Streptobacillus moniliformis*	0.00%	0.00%	0.11%	0.11%	0.72%	0.21%	0.03%	0.03%
*Proteobacteria*	*Caulobacteraceae*	*Brevundimonas diminuta*	0.00%	0.00%	2.69%	0.84%	0.41%	0.09%	22.48%	0.67%
		Order *Rhizobiales*	0.00%	0.00%	0.09%	0.03%	0.01%	0.00%	0.91%	0.09%
	*Bradyrhizobiaceae*	Family *Bradyrhizobiaceae*	0.00%	0.00%	0.73%	0.26%	0.18%	0.06%	1.23%	0.13%
	*Methylobacteriaceae*	Family *Methylobacteriaceae*	0.00%	0.00%	0.37%	0.12%	0.04%	0.01%	1.68%	0.08%
	*Sphingomonadaceae*	*Sphingobium* sp.	0.00%	0.00%	0.19%	0.06%	0.03%	0.01%	1.83%	0.10%
		*Sphingopyxis alaskensis*	0.00%	0.00%	0.27%	0.09%	0.04%	0.01%	2.37%	0.10%
	*Alcaligenaceae*	*Sutterella* sp.	2.85%	0.68%	0.03%	0.01%	0.00%	0.00%	0.03%	0.01%
	*Comamonadaceae*	*Delftia* sp.	0.00%	0.00%	0.22%	0.07%	0.04%	0.02%	1.75%	0.09%
	*Neisseriaceae*	Family *Neisseriaceae*	0.00%	0.00%	0.59%	0.40%	3.36%	0.80%	0.16%	0.04%
		*Conchiformibius* sp.	0.00%	0.00%	0.70%	0.44%	5.74%	1.40%	0.28%	0.19%
		*Neisseria animaloris*	0.00%	0.00%	0.07%	0.03%	0.63%	0.14%	0.00%	0.00%
	*Succinivibrionaceae*	*Anaerobiospirillum* sp.	3.14%	0.76%	0.03%	0.01%	0.00%	0.00%	0.05%	0.02%
		Order *Cardiobacteriales*	0.00%	0.00%	2.11%	0.71%	0.04%	0.04%	0.00%	0.00%
	*Enterobacteriaceae*	Family *Enterobacteriaceae*	0.90%	0.21%	0.04%	0.03%	0.00%	0.00%	0.14%	0.10%
	*Pasteurellaceae*	Family *Pasteurellaceae*	0.01%	0.00%	7.20%	4.88%	30.98%	4.30%	2.36%	0.89%
		*Aggregatibacter segnis*	0.00%	0.00%	0.49%	0.35%	4.22%	0.83%	0.00%	0.00%
		*Pasteurella multocida*	0.00%	0.00%	0.06%	0.02%	1.29%	0.59%	0.03%	0.01%
	*Moraxellaceae*	Family *Moraxellaceae*	0.00%	0.00%	10.31%	5.34%	0.12%	0.09%	0.09%	0.06%
		*Acinetobacter johnsonii*	0.00%	0.00%	1.91%	0.64%	0.34%	0.14%	19.81%	0.97%
		*Enhydrobacter* sp.	0.00%	0.00%	0.11%	0.06%	0.99%	0.19%	0.06%	0.02%
		*Moraxella* sp.	0.00%	0.00%	20.49%	6.36%	8.57%	3.93%	0.16%	0.08%
	*Pseudomonadaceae*	Family *Pseudomonadaceae*	0.00%	0.00%	5.23%	1.83%	0.61%	0.13%	29.63%	0.99%
		*Pseudomonas* sp.	0.01%	0.00%	0.33%	0.11%	0.06%	0.01%	2.73%	0.61%
*Tenericutes*	*Mycoplasmataceae*	*Mycoplasma* sp.	0.00%	0.00%	27.99%	8.13%	6.68%	4.59%	0.07%	0.04%

Mean and standard error of the mean (SEM) relative abundance of all taxa detected at greater than 0.50% mean relative abundance in at least one sample site, annotated to the level of phylum, family, and operational taxonomic unit.

**Fig 3:**
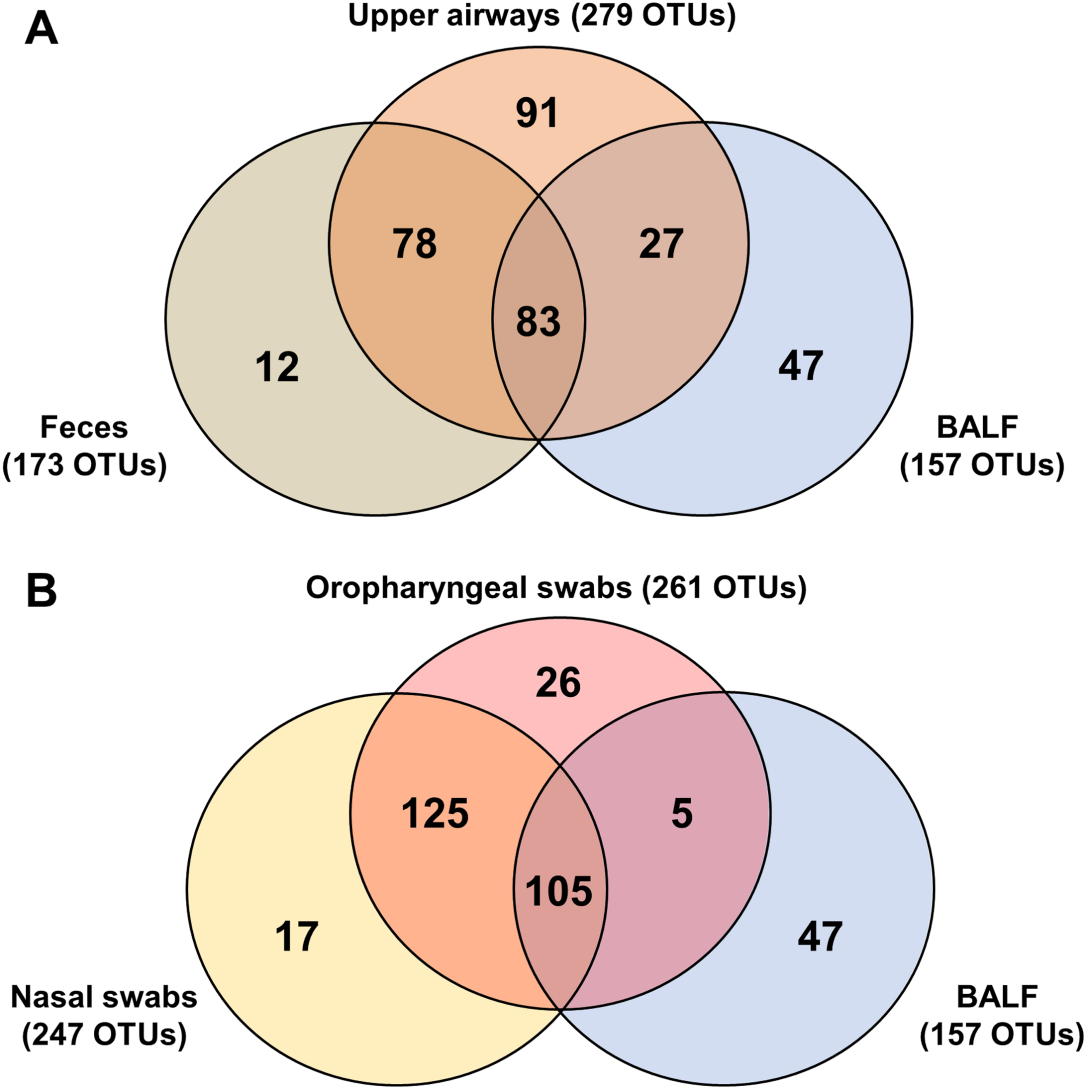
Distribution of all detected taxa between sample sites. Venn diagrams demonstrating the number of detected operational taxonomic units (OTUs) unique to each sample site, and shared between one or more sample site in feces, upper airways (nasal and oropharyngeal swabs combined), and bronchoalveolar lavage fluid (BALF) (**A**), or in nasal swabs, oropharyngeal swabs, and BALF (**B**).

This trend of selective colonization of the lower airways was also reflected in the patterns of co-occurrence of OTUs between the different sample sites. Of the 173 distinct OTUs detected in any of the fecal samples, 161 (93.1%) were also detected in one or both of the upper airway samples (Fig 3A). Conversely, 161 of the 279 OTUs (57.7%) identified in any of the upper airway samples was also detected in at least one of the fecal samples. In contrast, of the 157 OTUs detected in the lower airway (i.e., BALF) samples, 110 (70.1%) were also detected in the upper airway samples and there were no OTUs detected exclusively in both feces and the lower airways, suggesting a regional continuity of the microbial populations. A similar pattern emerged when upper airway samples were stratified between nasal and oropharyngeal samples (Fig 3B). Specifically, 230 of 247 OTUs (93.1%) detected in nasal samples were also detected in at least one of the oropharyngeal samples, and no OTUs were detected exclusively in nasal and lower airway samples. Collectively, the above data indicate that the airways harbor complex, largely aerobic microbial populations distinct from those found in the gastrointestinal tract, that the lower airways differ qualitatively from the upper airways, and that there exists a certain anatomic continuity in the microbiota as one descends from the nares to the oropharynx and then to the lung.

### Composition of Canine Lower Airway Microbiota Is Highly Conserved

**Fig 4:**
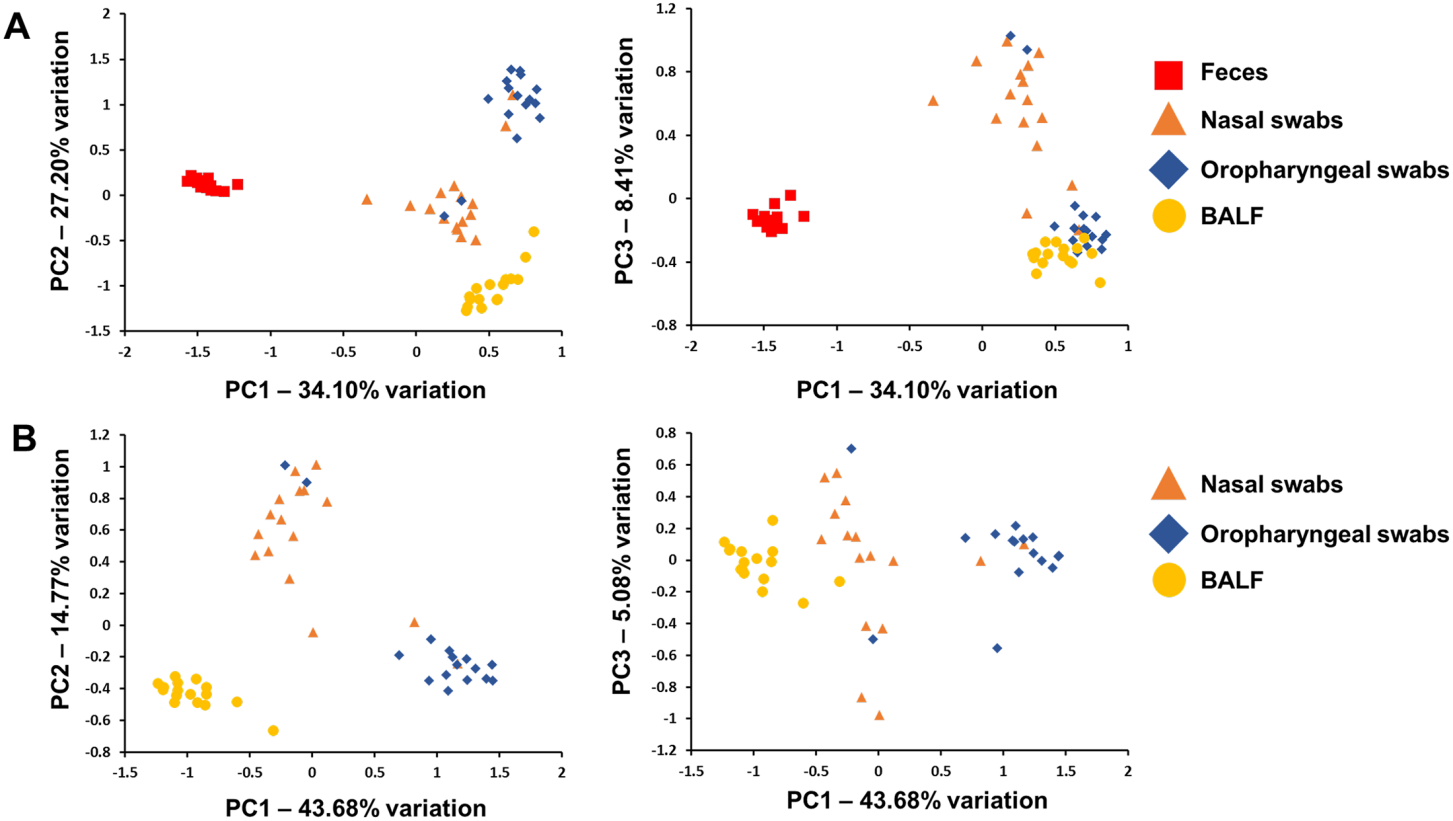
β-diversity as shown via principal component analysis. Unweighted principal component analysis of samples from all four sample sites (feces, nasal swabs, oropharyngeal swabs, and bronchoalveolar lavage fluid (BALF) (**A**) or only airway-associated samples (**B**). PC1 versus PC2 and PC1 versus PC3 in left and right panels respectively; legends at right.

To assess the overall relatedness of the bacterial populations present at each sample site, principal component analysis (PCA) was performed. PCA is a form of dimension reduction used to evaluate the β-diversity among samples, taking into account all detected OTUs. When samples from all sites were included in the analysis, principal component (PC) 1, accounting for 34.10% variation among the samples, demonstrated a complete separation of fecal microbial populations from the airway samples (Fig 4A). The airway samples clustered by sample site and separated along PC2 (27.20% variation) with slight overlap between the microbiota detected via nasal and oropharyngeal swabs. Interestingly, oropharyngeal swabs and BALF samples clustered together on PC3 (8.41% variation) indicating a certain degree of compositional similarity between those sites. As the stark contrast in composition between the fecal microbiota and that of the airways may obscure differences between the airway samples, additional plots were generated using only airway samples (Fig 4B). Again, samples clustered according to collection site and separated primarily along PC1 (43.68% variation).

**Fig 5:**
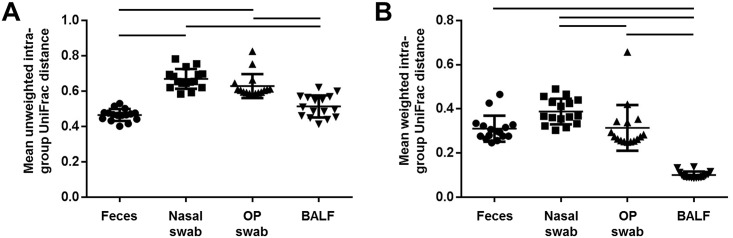
Compositional uniformity of samples from each site. Mean ± SEM unweighted (**A**) and weighted (**B**) intra-group UniFrac distance between each sample and all other samples collected from the same sample site. Bars indicate significant differences between groups (*p* < 0.05, Kruskal-Wallis one way ANOVA on ranks with multiple pairwise comparisons via Tukey test).

To statistically evaluate differences in compositional consistency of the samples collected from each site, data matrices of unweighted and weighted UniFrac distances were generated. UniFrac distances indicate the compositional similarity between two multivariate samples; unweighted UniFrac distances are determined based on the agreement between two samples regarding the presence or absence of all OTUs detected in either sample, while weighted UniFrac distances also take into account how closely samples agree with regard to the relative abundance of any shared taxa [10]. Specifically, a distance of 0 denotes identical composition while a distance of 1 denotes mutually exclusive bacterial populations. Thus, mean unweighted and weighted intra-group UniFrac distances can be used to assess how closely samples in a group agree with each other with regard to the presence and relative abundance, respectively, of OTUs detected in each sample. Considering first the unweighted intra-group UniFrac distance, fecal and BAL-obtained microbial populations were both more uniform than either the nasal or oropharyngeal samples (Fig 5A). There were no significant differences between the two upper airway samples, or between fecal and lower airway samples. Interestingly, the mean weighted intra-group UniFrac distance among the BALF samples was significantly lower than that for any of the other samples, indicating more uniform community structures in the lower airways than in upper airways or feces (Fig 5B). The mean weighted distance among the oropharyngeal swabs was also significantly lower than that of the nasal swabs. Collectively, these data demonstrate that the airways of healthy dogs are colonized by rich and diverse microbial communities with distinct differences between anatomic regions, and that the lower airways select for a uniform and consistent community structure composed primarily of bacteria in the phylum *Proteobacteria*.

### Predicted Metabolic Capacity of Airway Microbiota

While characterization of the microbial populations present at different levels of the airway provides useful information for certain potential future diagnostic and therapeutic avenues, an understanding of the metabolism of these microbial communities is also of great value. To determine the metabolic capacity of the upper and lower airway microbiota, the Phylogenetic Investigation of Communities by Reconstruction of Unobserved States (PICRUSt)[11] and HMP Unified Metabolic Analysis Network (HUMAnN)[12] software packages were applied to the current 16S rRNA amplicon dataset. Briefly, PICRUSt predicts the metabolic functional capacity of microbial communities characterized via 16S rRNA sequencing, based on the gene content of previously sequenced bacterial genomes, or the gene content of the most closely related genome sequences in the case of detected microbes which have not been fully sequenced. HUMAnN applies the non-parametric Kruskal-Wallis test to the gene content data and ranks the different gene categories by their effect size in each group of samples using a supervised linear discriminant analysis.

**Fig 6:**
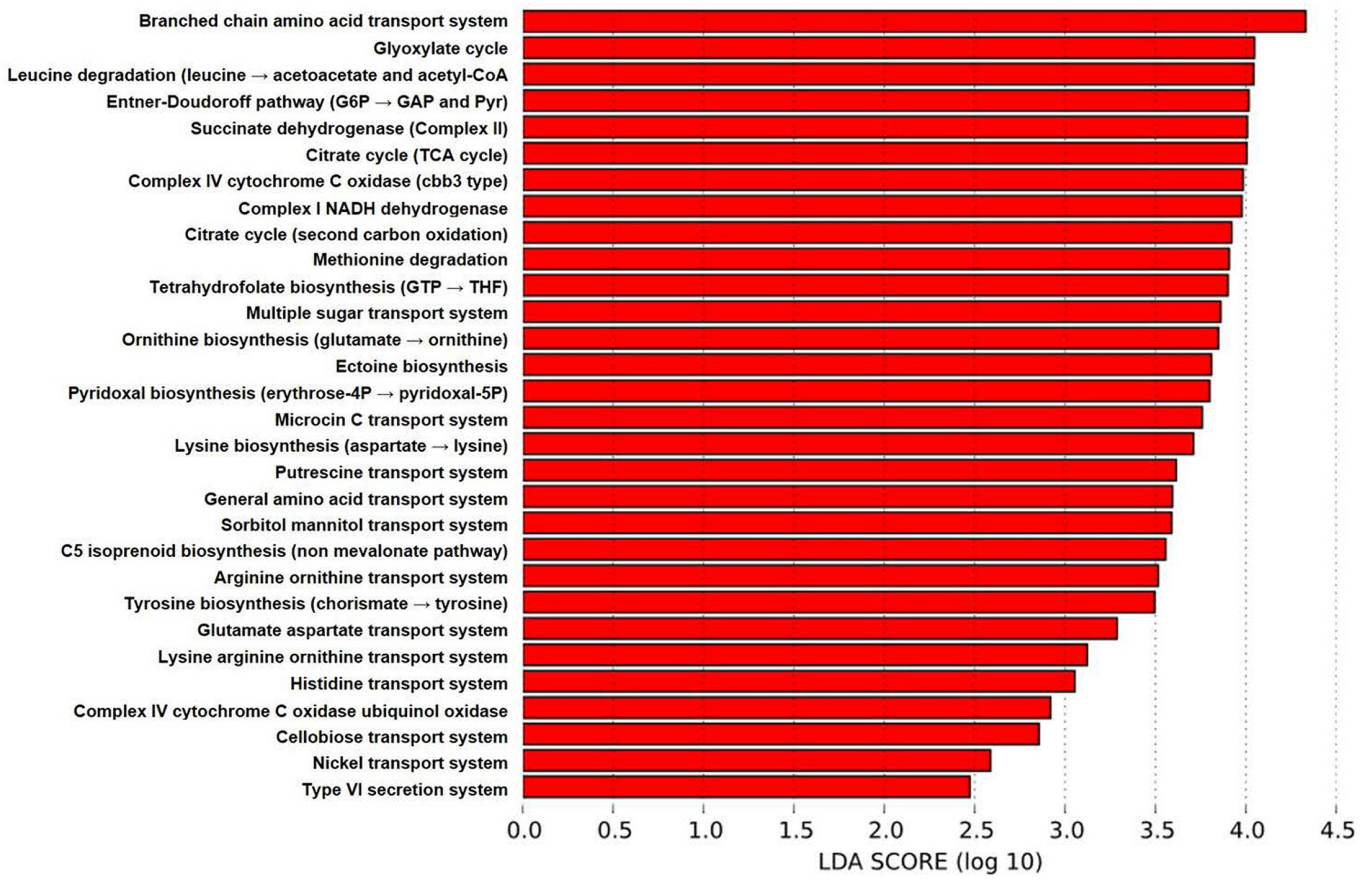
Gene content predicted at differential relative abundance between sample sites and at highest levels in BALF. Metabolic pathways identified using the Phylogenetic Investigation of Communities by Reconstruction of Unobserved States (PICRUSt) software and determined to be present at differential relative abundance between groups (based on Kruskal-Wallis ANOVA on ranks) and present at relatively greater abundance in bronchoalveolar lavage fluid (BALF) samples, ranked according to their effect size determined by linear discriminant analysis (LDA) score.

**Fig 7:**
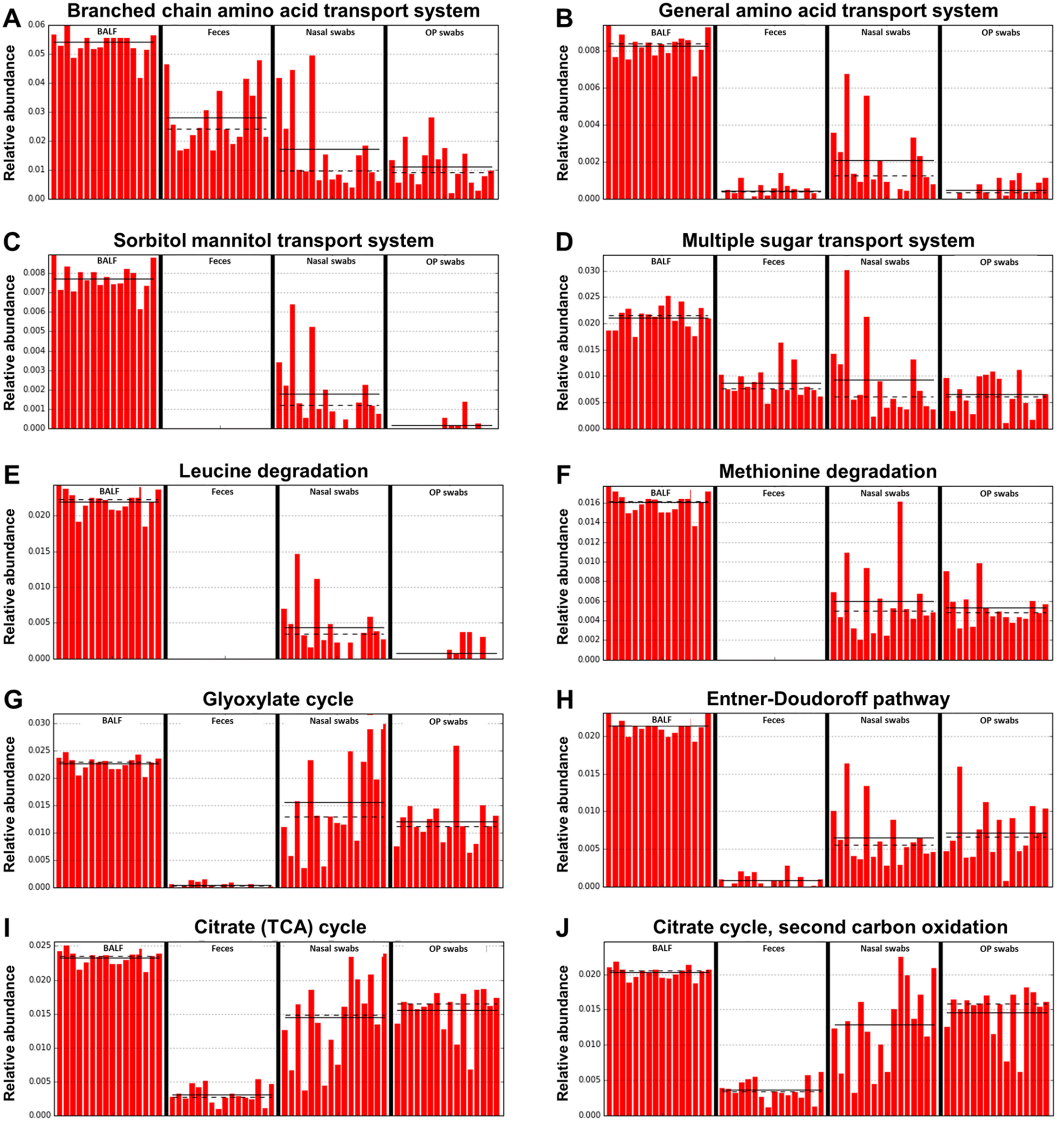
Relative abundance in each sample site of predicted gene content expressed at highest levels in BALF. Bar charts showing the relative abundance in each group of select pathways predicted to be enriched in bronchoalveolar lavage fluid (BALF) samples, including branched chain amino acid transport (**A**), general amino acid transport (**B**), sorbitol mannitol transport (**C**), multiple sugar transport (**D**), Leucine degradation (**E**), methionine degradation (**F**), the glyoxylate cycle (**G**) the Entner-Doudoroff pathway (**H**), the citrate cycle (**I**), and the second carbon oxidation of the citrate cycle (**J**); solid lines denote mean values within each group, dashed lines denote median values.

Using this approach, a total of 85 different gene functions, grouped according to KEGG (Kyoto Encyclopedia of Genes and Genomes) category [13, 14], were predicted to be present in significantly different abundance between groups, potentially serving as useful biomarkers of samples from one of the four different anatomic sites. Of note, the greatest number of characteristic gene categories (30 of 85) was identified in samples from the lower airways (Fig 6), several of which have putative functional interactions in nutrient transport or biosynthesis. Regarding uptake of nutrients, BALF-associated microbiota were predicted to possess more abundant gene content related to the transport of several carbon-containing compounds including branched-chain amino acids (i.e., leucine, isoleucine, valine) (Fig 7A), amino acids in general (Fig 7B), and several specific proteinogenic amino acids (e.g., arginine, glutamate, aspartate, lysine, and histidine); sorbitol/mannitol and other sugars (Fig 7C and 7D); and putrescine. There was also relatively greater abundance of genes related to nickel transport in BALF-associated microbiota, of note due to the identification of nickel as the preferred metal cofactor for the glyoxalase I enzyme in *Pseudomonas aeruginosa* [15].

Considering KEGG categories related to anabolic functions, gene content involved in the biosynthesis of tetrahydrofolate (a derivative of folate, vitamin B_9_), ornithine, ectoine (a protective osmolyte produced by multiple bacterial genera including *Pseudomonas* sp. and *Sphingopyxis alaskensis*)[16, 17], pyridoxal (vitamin B_6_), lysine, tyrosine, and C5 isoprenoids (via the non-mevalonate pathway) were all inferred to be present at relatively greater abundance in the BALF-associated microbiota. Catabolic pathways enriched in the BALF-associated microbiota included genes involved in the degradation of leucine and methionine (Fig 7E and 7F), resulting in the subsequent generation of acetyl-CoA from the former, and potentially S-adenosyl methionine from the latter.

Specific energy cycles predicted to be differentially expressed by airway bacterial communities, and BALF-associated microbes in particular, include the glyoxylate cycle (Fig 7G), the Entner-Doudoroff pathway (Fig 7H), and the citrate (tricarboxylic acid) cycle (Fig 7I and 7J).

Of note, the glyoxylate cycle results in the conversion of acetyl-CoA (generated from the predicted degradation of leucine) to succinate, and the downstream succinate dehydrogenase enzyme complex was also a predicted characteristic metabolic pathway in the BALF samples (Fig 6). Similarly, the citrate cycle, which also utilizes acetyl-CoA, results in the generation of CO_2_ and GTP and pathways involved in the biosynthesis of tetrahydrofolate from GTP were similarly identified in the BALF samples. Providing context for the presence of these metabolic pathways in microbial populations residing in a nutrient-poor environment, the glyoxylate and citrate cycles are related pathways identified in many aerobic microbes, which allow for energy production in the absence of glucose and other complex sugars.

**Fig 8:**
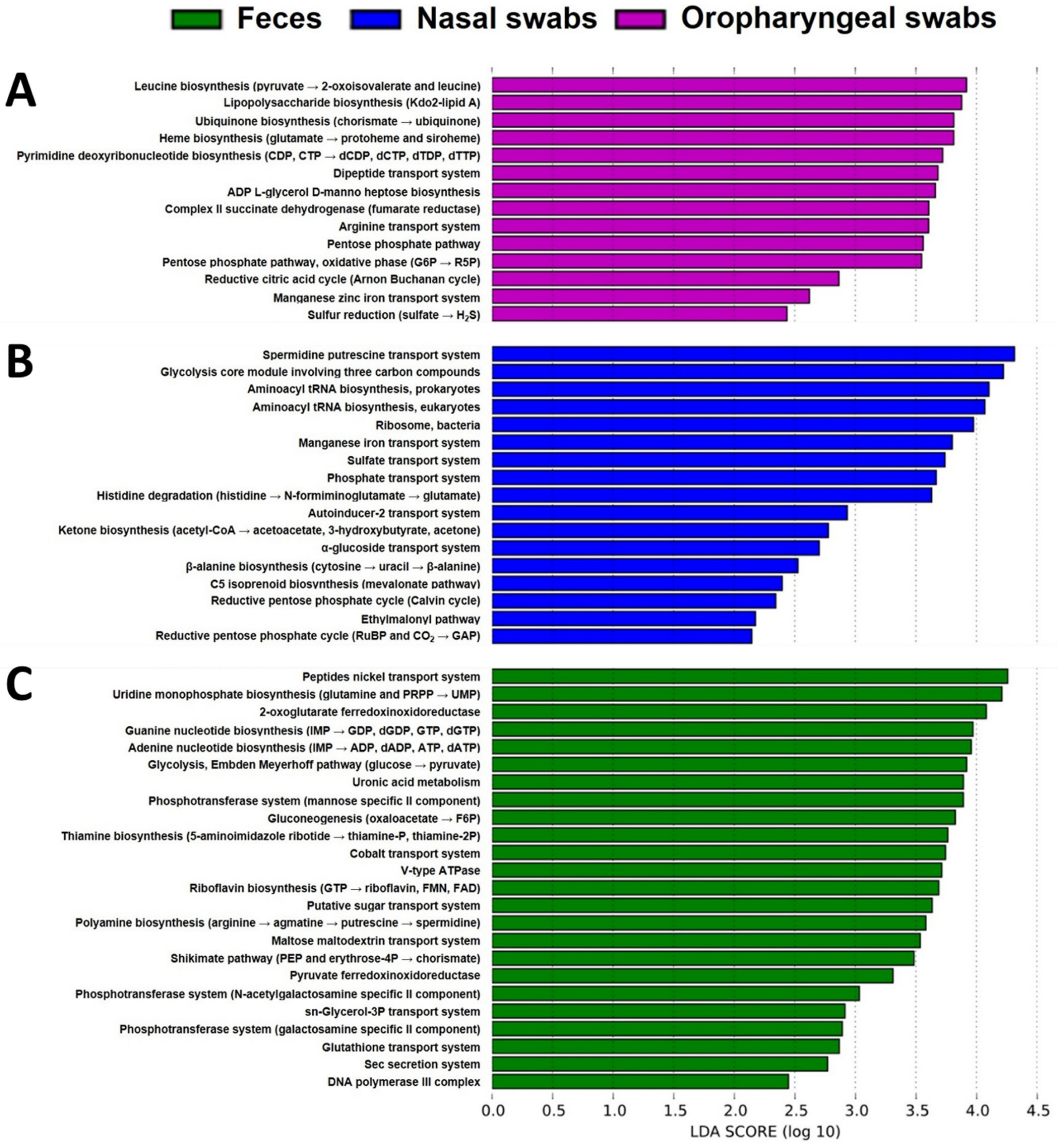
Gene content predicted at differential relative abundance between sample sites and at highest levels in feces, nasal swabs, and oropharyngeal swabs. Metabolic pathways identified using the Phylogenetic Investigation of Communities by Reconstruction of Unobserved States (PICRUSt) software and determined to be present at differential relative abundance between groups (based on Kruskal-Wallis ANOVA on ranks) and present at relatively greater abundance in oropharyngeal (**A**, purple bars), nasal (**B**, blue bars), and fecal (**C**, green bars) samples, ranked according to their effect size determined by linear discriminant analysis (LDA) score.

While there were also several gene categories predicted to be differentially expressed and present at greatest relative abundance in nasal and oropharyngeal microbiota, there were fewer than for BALF samples (Fig 8). Moreover, those predicted at a greater relative abundance in the nasal samples were present at marginally greater mean relative abundance in nasal samples when compared to the other airway regions; their identification via the Kruskal-Wallis ANOVA on ranks presumably being based on the relatively lower predicted gene content in fecal samples. Of those systems and pathways present in greater relative abundance in oropharyngeal samples, they primarily correlated with the abundance of family *Pasteurellaceae* in those samples. Considering the limited number of gene categories identified for that group and the resolution to only the taxonomic level of family, those data are difficult to interpret.

## Discussion

While the upper airways are routinely exposed to high bacterial loads derived from the oral and nasal cavities, the lungs, including bronchi, bronchioles, and alveoli, have historically been considered sterile in healthy individuals [18–20], a belief based on the lack of growth using traditional microbiological techniques and absence of histological evidence of microbes. However, repeated discrepancies in environmental and gastrointestinal samples between microbial densities observed via light microscopy and results of attempted cultivation, i.e., the great plate count anomaly, demonstrated that the majority of bacteria are refractory to *in vitro* cultivation [21]. The advent of molecular, culture-independent methods of interrogating microbial samples has revealed rich bacterial populations residing in myriad sites previously thought to be devoid of microbial life. The Human Microbiome Project, launched in 2008, was the first large-scale application of those methods to the human body, resulting in a relatively comprehensive view of the bacterial communities residing on external body surfaces, and various regions of the digestive and reproductive systems. The lack of inclusion of the respiratory tract was likely due to the aforementioned belief that the lower airways of healthy individuals harbored negligible microbial biomass, and also the invasive procedures required to collect a meaningful sample. Accordingly, many of the initial studies of airway microbiota employing molecular techniques examined only patients diagnosed with a specific disease (and for whom a BALF sample was collected as part of their standard medical care) [22], or included controls affected with pulmonary disease other than the disease of specific interest [23]. It is only within the last decade that a handful of reports have emerged documenting the existence of bacteria within the lungs of healthy individuals [2, 3, 5, 7]. There are few, if any, reports of the normal airway microbiota found in domestic or research animal species.

Differences in the community composition and population dynamics are to be expected between gut and lung microbiota based on unique local environmental features and anatomic differences [24]. In the gut, movement of microbes is unidirectional with chemical and physical barriers separating compartments; in the lung, microbes move in a bidirectional fashion with air and mucus, and no physical barrier separates the larynx from the alveoli. Additionally, within the respiratory tract, variations in temperature, high oxygen concentration, bacteriostatic surfactant [25, 26], and markedly lower bacterial density (leading to differences in quorum sensing) are other factors which contribute to a divergent microbiota. In the dog, for the first time, we have documented that the gut, nasal passages, oropharynx, and lower airways each have complex microbiota, with a degree of regional continuity with deeper descent into the airways. As in healthy humans [3, 27], healthy dogs have the highest bacterial signal levels from the gut and oropharynx, with lower numbers of sequence per sample detected in the nasal passages and lung.

Unexpectedly, BALF in healthy dogs had equivalent richness to feces and nasal swabs. Furthermore, canine BALF had the most uniform community structure of all anatomic regions examined. While multiple studies in humans have documented clear differences between the lung microbiome in health versus respiratory disease [5, 7], the healthy human lung seemingly lacks a consistent and unique microbiota [3]. Having dogs of the same breed/genetic background co-housed in a stable environment and eating the same diet may have contributed to the uniformity of the BALF microbiota, although it would not explain the more varied community structures from other sample sites.

Just as in studies of the gut microbiota [28], the preponderance of research on human airway microbiota has produced correlative data, leading to uncertainty as to whether compositional differences observed between experimental groups represent a cause or effect of the disease under study. This is largely due to ethical considerations, as well as the extended time course over which many diseases develop. Thus, comparative medicine, i.e., the use of appropriate animal model systems to study disease mechanisms and evaluate diagnostic assays and therapeutic interventions for possible use in humans, is a vital component of research in the field of metagenomics. The need for such tools was specifically identified in the recent Report of the Fast-Track Action Committee on Mapping the Microbiome, produced by the National Science and Technology Council. The current data support the use of dogs as a potential model species for prospective, controlled studies of the influence of airway microbiota on health and disease susceptibility, and the efficacy of potential therapies on resolution of airway disease. Not only are there induced respiratory disease models in research dogs, but pet dogs spontaneously develop a variety of respiratory disorders which mimic clinicopathologic features of human disease (e.g., lung cancer, inflammatory airway disease, aspiration pneumonia, infectious pneumonia, etc.).

While there is variability in the composition of the lower airway microbiota detected in human studies, possibly due to differences in the study population and methodologies, certain characteristic taxa have been identified in multiple studies. Specifically, *Prevotella*, *Streptococcus*, *Acinetobacter*, *Veillonella*, *Pseudomonas*, *Fusobacterium*, *Neisseria*, and *Porphyromonas* have been identified as prominent genera in BALF or cytology brush samples of healthy humans, albeit at differing relative abundances. Notably, all of these taxa were detected in the majority of canine BALF samples in the present study. Specifically, *Prevotella copri* was detected in 14/16 BALF samples (mean 0.092% overall); *Streptococcus* sp. in 16/16 BALF samples (mean 0.848%); *Acinetobacter johnsonii* and *Acinetobacter* sp. in 16/16 and 9/16 BALF samples (mean 19.814% and 0.064% respectively); *Veillonella parvula* in 14/16 BALF samples (mean 0.079%); *Pseudomonadaceae* and *Pseudomonas* sp. in 16/16 BALF samples (mean 29.628% and 2.733% respectively); *Fusobacterium* in 16/16 BALF samples (mean 0.372%); *Neisseriaceae* in 14/16 BALF samples (mean 0.156%); and *Porphyromonas* sp. in 10/16 BALF samples (mean 0.442%). Conversely, *Brevundimonas* sp., one of the dominant OTUs detected in canine BALF, has been identified only rarely in human BALF samples [2, 3, 5]. Another taxon found in several human studies but not specifically found in the present dataset is *Haemophilus* sp. although this could be due to the resolution afforded by the primer set. Taken together, the current findings suggest that the lung microbiota of healthy dogs is similar to that of healthy humans, at least to the taxonomic level of genus. That being said, it should be remembered that the current subjects were purpose-bred research dogs housed in a controlled environment and the composition of the airway microbiota in dogs living as companion animals may show greater variability.

Regarding the upper airway microbiota, Hilty et al. reported abundant *Corynebacterium* sp., *Staphylococcus* sp., and *Moraxella* sp. in nasal samples, abundant *Prevotella* sp. in oropharyngeal samples, and high levels of *Streptococcus* sp., *Veillonella* sp., and *Neisseria* sp. in both regions [7]. In agreement with the present data, *Acinetobacter* sp. and *Pseudomonas* sp. were also detected in the upper airways but rare. Alternatively, Charlson et al. were unable to detect consistent differences between the composition of upper and lower airways in their study of 6 healthy individuals, and concluded that the lower airways of healthy humans were largely devoid of a distinctive bacterial population, and any microbial DNA detected there was a result of microaspiration from the upper airways or artifact [3]. While the data reported here support their findings of extremely low biomass in the lower airways and do not obviate microaspiration as the source of lower airway microbes, the compositional consistency of the BALF-associated microbiota reported above indicate that these are true, colonizing microbial communities. As noted elsewhere [24], it would be remarkable if a warm, moist, mucosal surface in such close proximity to the oral and nasal cavities was unable to support some degree of colonization by microbes adapted to such an environment. Of note, a substantially larger volume of BALF was used as the source of DNA in the current study compared to that used in Charlson et al. (mean of 4.26 mL versus 1.8 mL), resulting in greater coverage (mean of 5706 sequences per canine BALF sample as opposed to roughly half that in the human study [3]). Thus, it is possible that detection of bacterial DNA in the lower airways was enhanced in the present study through use of a larger volume of BALF.

Clinically, the consistent detection of *Pseudomonaceae*, *Pseudomonas* sp., and *Acinetobacter johnsonii* in BALF samples is of particular interest in the context of nosocomial infections, including ventilator-assisted pneumonia. While the ability of such microbes to produce biofilms and express and transfer antimicrobial resistance genes are clearly associated with their virulence [29, 30], the current data suggest that they might be part of a pre-existing resident airway microbiota that, under specific pressures such as antibiotic treatment, allows the aberrant proliferation of individual community members, akin to *Clostridium difficile* overgrowth of the GI tract. While less common, *Brevundimonas diminuta* has also been associated with nosocomial infections and resistance to certain drug classes [31].

While the taxonomy of the bacterial communities present in the canine airways is of great interest, the use of dogs as a model species for translational research will require information related to the metabolism of said communities. Comparison of the gene categories predicted in the bacterial populations detected at each site indicated that BALF-associated microbiota may be particularly adept at the uptake of amino acids and various sugars, including sorbitol and mannitol. Mannitol, an isomer of sorbitol, is a common energy and carbon storage molecule produced by a wide variety of microorganisms, prokaryotes included [32]. Thus, the high predicted abundance of this system may represent a mechanism of energy conservation by microbes colonizing an energy-sparse environment.

Interestingly, the BALF-associated microbiota also appears to possess abundant genetic content related to the metabolism of C_2_ and C_3_ carboxylic acids. The tricarboxylic acid (TCA) cycle, also known as the citrate cycle or Krebs cycle, is a central metabolic pathway utilized by all aerobic organisms, generating energy in the form of ATP and GTP, and multiple intermediate compounds that can serve as substrates for biosynthesis of amino acids, fatty acids, and cholesterol. That the citrate cycle may indeed be utilized by the lower airway microbiota to generate precursors to amino acids is supported by the fact that the transamination reaction needed to transform α-keto-acids into usable amino acids requires the cofactor pyridoxal, the synthesis of which was also predicted to be preferentially abundant in the BALF-associated microbiota. Regarding the metabolism of C_2_ carboxylic acids, many bacteria rely on the glyoxylate pathway to generate acetyl-CoA as an energy source for biosynthesis. Succinate, a metabolic product of this pathway, can be converted into carbohydrates via combination of the citrate cycle and gluconeogenesis, thus providing a metabolic versatility to organisms possessing the glyoxylate pathway machinery.

In summary, the current data suggest that the upper and lower canine airways are colonized by rich microbial populations that differ between the nasal cavity, oropharynx, and lungs, and that the microbiota present in the lungs assumes a relatively uniform composition, despite its low biomass. The consistent composition of the lower airway microbiota is presumably due to the low level of available resources, and the microbes preferentially possess genetic content that allows for the uptake and efficient metabolism of several basic carbon-containing compounds that can be converted into other essential compounds or energy sources. Future metatranscriptomic, metabolomic, and proteomic studies are needed to confirm the predicted metabolic pathways identified in the current study. Moreover, additional work is needed to assess the metabolism of human airway microbial populations and interrogate differences between healthy and diseased airways.

## Materials and Methods

### Ethics Statement

All studies were performed in accordance with the recommendations put forth in the Guide for the Use and Care of Laboratory Animals, and were approved by the University of Missouri Institutional Animal Care and Use Committee (MU IACUC protocol #7349).

### Dogs

Dogs were all healthy, purpose-bred intact female adult beagles between 2 and 8 years of age (mean 3.8 years), with a mean ± standard deviation body weight of 11 ± 1.4 kg, purchased from Sinclair Research. All dogs were on site for a minimum of 35 days prior to sample collection. Dogs were housed alone or in pairs (depending on temperament) in tender foot pens, fed Purina Lab Diet 5006 and given *ad libitum* access to clean drinking water. To minimize risk of aspiration, dogs were fasted for at least 12 hours prior to induction of anesthesia. Dogs were confirmed to be healthy and free of respiratory disease via absence of respiratory clinical signs, normal physical examinations, non-inflammatory BALF cytology (mean ± SEM percentage of nucleated cells: alveolar macrophages 94.3 ± 0.5%; neutrophils 3.5 ± 0.3%; eosinophils 1.1 ± 0.3%) and absence of lung lesions on histopathology. Following collection of all samples and prior to recovery from anesthesia, dogs were humanely subjected to euthanasia via an overdose of injected pentobarbital according to the 2013 AVMA Guidelines for the Euthanasia of Animals, as the endpoint of an unrelated terminal study.

### Sample Collection

Fecal samples were collected via manual evacuation using a gloved finger. Dogs were anesthetized and intubated using a sterile endotracheal tube. A moistened swab was inserted midway between the tip of the nose and the medial canthus of the eye, rotated vigorously and removed. With the dogs’ mouth held open and tongue pulled forward, a second moistened swab was used to vigorously rub the back of the oropharynx, taking care to avoid the oral cavity. To obtain the lower airway sample, the dog was positioned in lateral recumbency and a sterile 8 French red rubber catheter was passed through the lumen of the sterile endotracheal tube until firm resistance was felt. A 20 mL aliquot of sterile saline was instilled and gently aspirated using gentle manual suction, retracting the catheter slightly as needed to remove the saline. All samples were placed on ice for transport to the laboratory.

### DNA Extraction

All DNA was extracted using a manual precipitation protocol as previously described [33]. For extraction of fecal DNA, a small piece of feces (approximately 25 mg) was placed in a sterile 2 mL round-bottom tube containing a 0.5 cm diameter stainless steel ball bearing and 800 μL lysis buffer adapted from Yu et al. (500 mM NaCl, 50 mM Tris-HCl pH 8.0, 50 mM EDTA, and 4% sodium dodecyl sulfate) [34]. For nasal and oropharyngeal samples, swabs were placed in 800 μL lysis buffer and agitated thoroughly for several minutes to allow for lysis of bacteria. For extraction of BALF samples, recovered material was centrifuged at 5000 × g for 10 minutes, followed by removal of supernatant and resuspension in 800 μL lysis buffer. All samples were then incubated at 70°C for 20 minutes with periodic vortexing, and centrifuged at 5000 × g for 5 minutes. Two hundred μL of 10 mM ammonium acetate was added to the supernatants and, following brief vortexing, samples were incubated on ice for 5 minutes, and then centrifuged at 5000 × g for 15 minutes, at room temperature. The supernatant was again transferred to a new Eppendorf tube and one volume of chilled isopropanol was added. Samples were incubated on ice for 30 minutes and then centrifuged at 16,000 × g for 15 minutes, at 4°C. DNA pellets were washed with 70% ethanol and resuspended in 150 μL Tris-EDTA (10 mM Tris and 1 mM EDTA), followed by addition of 15 μL of proteinase K and 200 μL of AL Buffer (DNeasy Blood and Tissue kits, Qiagen). Samples were incubated at 70°C for 10 minutes and 200 μL of 100% ethanol was added to the tubes. Samples were then mixed by gentle pipetting and the contents transferred to a spin column from the DNeasy kit. The DNA was purified following the manufacturer’s instructions and eluted in 200 μL EB buffer. DNA concentrations were determined via fluorometry (Qubit dsDNA BR assay, Life Technologies, Carlsbad CA) and samples were stored at -20°C until PCR and sequencing.

### 16S rRNA Library Preparation and Sequencing

Library construction and sequencing was performed at the University of Missouri DNA Core facility. DNA concentration of samples was determined fluorometrically and all samples yielding greater than 100 ng total DNA were normalized to 3.51 ng/μL for PCR amplification. Bacterial 16S rRNA amplicons were generated via amplification of the V4 hypervariable region of the 16S rRNA gene using single-indexed universal primers (U515F/806R) flanked by Illumina standard adapter sequences and the following parameters: 98^°^C^(3:00)^+[98^°^C^(0:15)^+50^°^C^(0:30)^+72^°^C^(0:30)^] × 25 cycles +72^°^C^(7:00)^. Amplicons were then pooled for sequencing using the Illumina MiSeq platform and V2 chemistry with 2×250 bp paired-end reads, as previously described [35].

### Informatics Processing

Assembly, binning, and annotation of DNA sequences was performed at the MU Informatics Research Core Facility. Briefly, contiguous DNA sequences were assembled using FLASH software [36], and culled if found to be short after trimming for a base quality less than 31. Qiime v1.8 [37] software was used to perform *de novo* and reference-based chimera detection and removal, and remaining contiguous sequences were assigned to operational taxonomic units (OTUs) via *de novo* OTU clustering and a criterion of 97% nucleotide identity. Taxonomy was assigned to selected OTUs using BLAST [38] against the Greengenes database [39] of 16S rRNA sequences and taxonomy. Principal component analyses were performed using ¼ root-transformed OTU relative abundance data via a non-linear iterative partial least squares (NIPALS) algorithm, using an open access Excel macro available from the Riken Institute (http://prime.psc.riken.jp/Metabolomics_Software/StatisticalAnalysisOnMicrosoftExcel/index.html). The PICRUSt package [11] was used to predict functional capacity of each sample, and the HUMAnN package [12] was used to predict gene categories present at differential levels between sample sites.

### Statistical Analysis

Statistical analysis was performed using Sigma Plot 12.3 (Systat Software Inc., Carlsbad, CA). Differences between sample collection sites in total DNA concentration, microbial richness, and mean intra-group UniFrac distances were determined using Kruskal-Wallis ANOVA on ranks with post hoc comparisons via Tukey test. Results were considered statistically significant for *p* values ≤ 0.05.

## Supporting Information

S1 Fig Coverage and detected richness of all samples. Mean ± standard error of the mean (SEM) coverage, i.e., number of sequences per sample, detected in DNA extracted from feces, nasal swabs, oropharyngeal (OP) swabs, and bronchoalveolar lavage fluid (BALF) collected from 16 intact adult female dogs (**A**). Mean ± SEM richness, i.e., number of unique operational taxonomic units (OTUs) detected in the same samples (**B**). Bars denote significant (*p* ≤ 0.05) differences as determined using Kruskal-Wallis ANOVA on ranks with post hoc comparisons via Tukey test.
(TIF)

## Abbreviations

BALF: bronchoalveolar lavage fluid
OTU: operational taxonomic unit
PCA: principal component analysis
