# Supplementary figures and images for: Composition and Predicted Metabolic Capacity of Upper and Lower Airway Microbiota of Healthy Dogs in Relation to the Fecal Microbiota
Source: PLoS One. 2016 May 2;11(5):e0154646. doi: 10.1371/journal.pone.0154646 (PMC4852910; doi:10.1371/journal.pone.0154646)

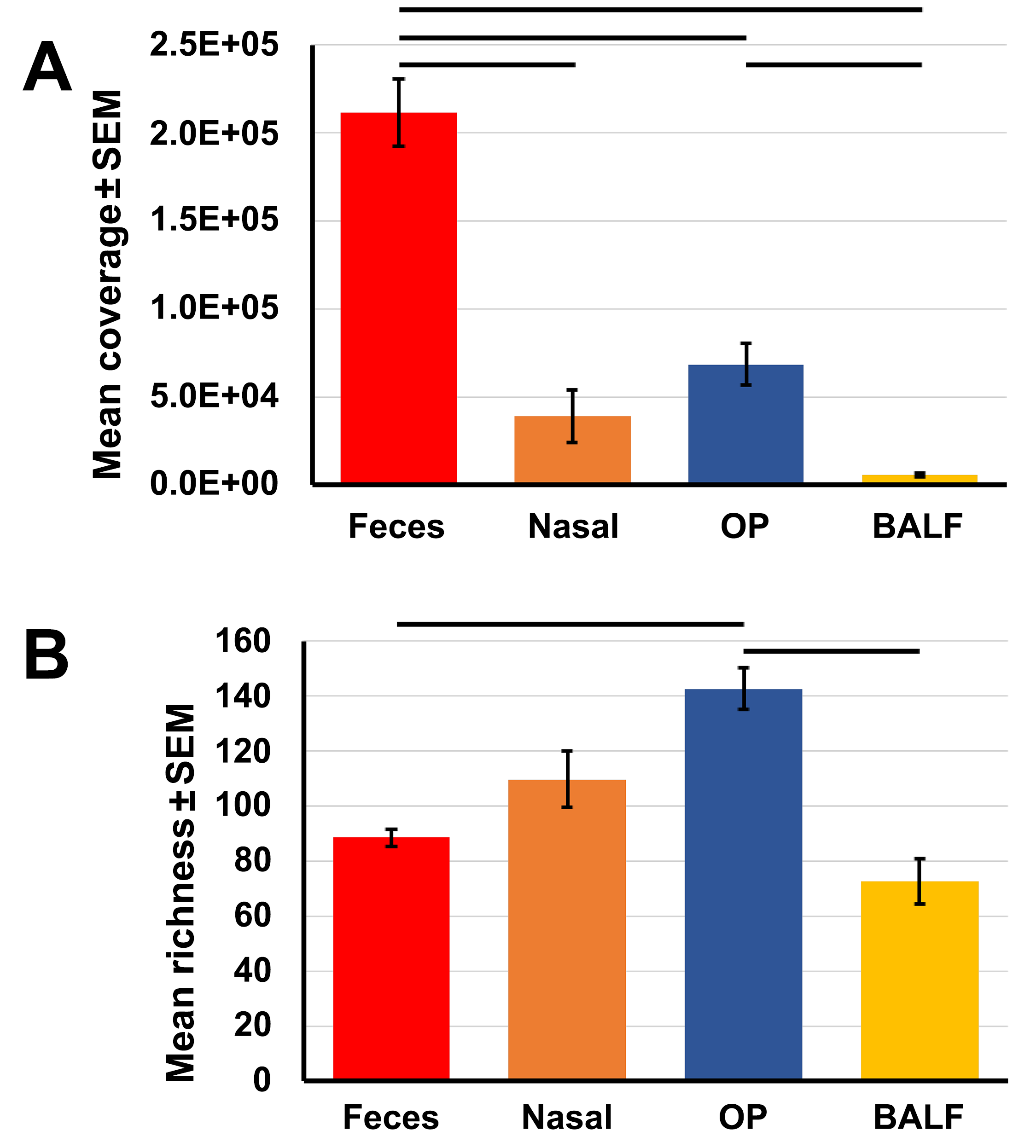

Supplement: S1 Fig — Mean ± standard error of the mean (SEM) coverage, i.e., number of sequences per sample, detected in DNA extracted from feces, nasal swabs, oropharyngeal (OP) swabs, and bronchoalveolar lavage fluid (BALF) collected from 16 intact adult female dogs (A). Mean ± SEM richness, i.e., number of unique operational taxonomic units (OTUs) detected in the same samples (B). Bars denote significant (p ≤ 0.05) differences as determined using Kruskal-Wallis ANOVA on ranks with post hoc comparisons via Tukey test. (TIF) [file pone.0154646.s001.tif]
